# Improvement of Pain Symptoms in Musculoskeletal Diseases After Multimodal Spa Therapy in the Austrian Gastein Valley—A Study Based on Longitudinal Registry Data

**DOI:** 10.3389/ijph.2023.1605931

**Published:** 2023-06-08

**Authors:** Antje van der Zee-Neuen, Julia Fuchs, Sonja Wildburger, Martin Gaisberger, Margreet Kloppenburg, Antonella Fioravanti, Tanja Stamm, Martin Offenbaecher, Rudolf Radlmueller, Wolfgang Foisner, Bertram Hoelzl, Markus Ritter

**Affiliations:** ^1^ Center for Physiology, Pathophysiology and Biophysics, Institute for Physiology and Pathophysiology, Paracelsus Medical University, Salzburg, Austria; ^2^ Gastein Research Institute, Paracelsus Medical University, Salzburg, Salzburg, Austria; ^3^ Center for Public Health and Healthcare Research, Paracelsus Medical University, Salzburg, Austria; ^4^ Center for Physiology, Pathophysiology and Biophysics, Institute for Physiology, Pathophysiology and Biophysics, Nuremberg, Germany; ^5^ Ludwig Boltzmann Institute for Arthritis and Rehabilitation, Vienna, Austria; ^6^ Department of Rheumatology, Leiden University Medical Center (LUMC), Leiden, Netherlands; ^7^ Department of Clinical Epidemiology, Leiden University Medical Center (LUMC), Leiden, Netherlands; ^8^ Azienda Ospedaliera Universitaria Senese, Siena, Italy; ^9^ Institute for Outcomes Research, Center for Medical Data Science, Medical University of Vienna, Vienna, Austria; ^10^ Gastein Healing Gallery, Bad Gastein, Austria; ^11^ Alpentherme Gastein Gesundheitszentrum, Bad Hofgastein, Austria; ^12^ Department of Internal Medicine, Landesklinik St. Veit im Pongau, SALK, Paracelsus Medical University, Salzburg, Austria; ^13^ Kathmandu University School of Medical Sciences, Dhulikhel, Nepal

**Keywords:** longitudinal study, musculoskeletal diseases, rheumatic diseases, radon spa, pain

## Abstract

**Objective:** The study aim was to investigate the course of pain in rest and motion in seven different rheumatic diseases (RMD), prior and after multimodal spa therapy including low-dose radon treatment and at 3-, 6-; and 9-month follow up.

**Methods:** Complete data from the radon indication registry including information on 561 subjects with RMD were analysed to explore the association of timepoint of measurement with pain in rest and motion. For this purpose, linear regression models adjusted for RMD-type, age, sex and body mass index (BMI) were applied.

**Results:** The mean age of the sample was 55 years, the average body mass index was 26.8, and 275 subjects were women. Pain scores were significantly improved at all-time points compared to baseline. Pain courses were different for each RMD with the largest improvement seen in fibromyalgia.

**Conclusion:** Timing spa facility visits according to RMD-specific pain courses may result in sustained pain reduction.

## Introduction

Rheumatic musculoskeletal diseases (RMD) are among the most prevalent disorders worldwide. RMD is an umbrella term that refers to several conditions affecting the musculoskeletal system (i.e., joints, tendons, muscle, ligaments, bones) among which osteoarthritis (OA), rheumatoid arthritis (RA), ankylosing spondylitis (AS), fibromyalgia (FBM), psoriatic arthritis (PsA) and low back pain (BP). Globally, approximately 1.71 billion individuals suffer from a RMD. These diseases are responsible for the largest proportion (17%) of global years lived with disability (YLDs) equalling approximately 149 million YLDs [1, 2]. While RMD are heterogeneous in terms of their cause and course, common symptoms include disability, decreased quality of life (QoL) and the presence of musculoskeletal pain in rest and/or motion [3–5].

Depending on the type of RMD, treatment options encompass non-pharmacological and/or pharmacological measures aiming at the reduction of pain and improvement of physical functioning as well as the stabilisation of symptoms. For some diseases, surgical measures are available [6–8]. Pharmacological treatment options are wide and recent development has resulted in cost-effective pharmacological treatment options [8]. Such treatment may employ corticosteroids, disease-modifying anti-rheumatic drugs (DMARDs), nonsteroidal anti-inflammatory drugs (NSAIDs), paracetamol or other oral or topical medication to control symptoms like inflammation, pain and functional disability. However, despite their clear benefits they also have a broad bandwidth of adverse side effects. For example, NSAIDs, often prescribed for patients with RMD may cause gastrointestinal, renal, hepatic, cardiovascular, cerebral and pulmonary complications, which are potentially lethal to patients. Accordingly, the uptake of paracetamol in high or sustained doses (like in the treatment of pain in OA patients) is associated with an increased risk of severe side effects including an increased risk of mortality and gastro-intestinal, renal and cardiovascular adverse events. Such side effects result in hospital admissions adding to the socio-economic burden of RMD [9–13].

Alternative or complimentary non-pharmacological treatment options have the potential to alleviate the burden by reducing the need for pharmaceutical uptake. In Europe, balneotherapy is among the most commonly prescribed non-pharmacological complementary therapies for different RMD (14). Balneotherapy is frequently described as part of health resort medicine (HRM) and may be considered a valid treatment option along with other HRM treatments like hydrokinesitherapy (i.e., physiotherapy in thermal water) and more generalized spa therapy which consists of a multimodal treatment approach (including exercise, massages, hydrokinesitherapy, mild hyperthermia interventions, etc.) [14–16].

Spa therapy including low-dose radon treatment is a specific sub-group of spa therapy and has shown to be beneficial in the improvement of various symptoms related to RMD, including pain, QoL and physical functioning [17, 18]. The effectiveness of this type of therapy in the alleviation of pain appears to be comparable or superior to RMD spa therapy without radon treatment. For example, a Cochrane review by Verhagen et al. summarized trials on the effectiveness of balneotherapy for RA and concluded that there is no statistical difference in pain frequency 3 months after balneotherapy with or without radon, yet that there is some benefit of additional radon when evaluating pain frequency 6 months after treatment. On the same line, Gaisberger et al. [19] found significant reductions in self-reported knee pain in patients with knee-OA until 6 months after multimodal spa therapy with and without radon treatment. Similarly, Winklmayr et al. [20] found statistically comparable improvements in somatic complaints and bone metabolism regulation of adults between 50 and 65 years when comparing participants that followed a physical activity regimen for osteoporosis prevention including baths in either radon thermal water or radon free thermal water. Polshchakova et al. [21] observed superiority of radon balneotherapy compared to placebo baths in the reduction of pain and movement difficulties in patients with osteochondrosis of the spine. Long lasting pain reduction until 30 weeks after initiation of radon spa therapy was also reported for patients suffering from chronic pain due to RMD of the spine (*n* = 38), the joints (*n* = 32), the spine and the joints (*n* = 22) or FBM (*n* = 8) [22].

However, the course of the clinical effects of spa therapy including low-dose radon treatment, like the reduction of pain, may be very different across individual disease types. Yet, research usually focusses on one or a small selection of RMD. One previous study investigated the effect of spa therapy including low-dose radon on pain relief across various diseases in a controlled setting and found superiority of radon baths against placebo baths [23]. The relevant outcomes of this study advocate the evaluation of clinical outcomes in a real-life setting in order to provide medical decision makers and insurance companies with the required information for decisions on treatment intervals and frequency as potential differences in the course of improvement in pain might justify individualized timing of therapy.

In the current study, we therefore aimed 1) to explore the course of pain severity after multimodal spa therapy including low-dose radon treatment in a sample of patients with various RMD and 2) to predict the course of pain severity separately for each individual disease.

We hypothesized that 1) multimodal spa therapy including low-dose radon treatment is beneficial in reducing pain in rest and motion in patients with RMD in an observational, real-world setting and 2) the course of pain severity following multimodal spa therapy including low-dose radon treatment is different for each disease.

## Methods

A longitudinal analysis of prospectively collected register data from the ongoing “Radon indication registry for the assessment of pain reduction, increase of quality of life and improvement in body functionality throughout low-dose radon hyperthermia therapy” (registration ID ISRCTN67336967; https://doi.org/10.1186/ISRCTN67336967, short radon registry) in the valley of Gastein in Austria was conducted.

Data for the radon registry is collected from participants directly before (baseline), directly after as well as 3, 6 and 9 months after multimodal spa therapy including low-dose radon treatment by 4 participating spa facilities in the Austrian valley of Gastein.

Based on prescriptions by local physicians, the intervention consists of multimodal, non-pharmacological spa therapy including low-dose radon treatment in the valley of Gastein in the Austrian Alps. The average intervention consists of several different treatments including physical exercise, massages, lymphatic drainage, mud therapy, ergometry, progressive muscle relaxation, trainings and consultations for back pain prevention, anti-smoking and healthy nutrition as well as medical examination and lectures. All patients also receive low-dose radon balneotherapy and/or low-dose radon speleotherapy. The balneotherapeutic radon treatment consists of bathing in water (∼37°C) with low activity of radon (average 707.57 (SD 233.27) Bq/L measured by liquid scintillation, Triathler™ LSC Hidex). The thermal water consists of (in mg/kg water): Na^+^ 80.01; K^+^ 5.71; Li^+^ 0.27; Ca^2+^ 19.84; Mg^2+^ 0.75; HCO_3_^–^ 57.91; Cl^-^ 24.96; F^−^ 5.61; SO_4_^2–^ 130.67; H_2_SiO_3_ dissociated 46.16/colloidal 8.17; H_3_BO_3_; CO_2_ 6.87. Trace elements are (in mg/L water): Hg 0.0008; As <0.02; Pb 0.08; Cr < 0.01; Se < 0.05; Cd < 0.01; CN^–^ <0.01 [24]. A balneotherapy intervention including low-dose radon consists of approximately 10 baths with a duration of 20 min.

The speleotherapeutic radon treatment consists of relaxation in the healing gallery of Gastein (a former mine, dug in search for gold) for an average time of 60 min on alternate days (i.e., an average of 11 speleotherapy sessions). The facility is located in moderate altitude (1,270 m above sea level) with a low atmospheric activity of radon (on average 44 kBq/m^3^ air, as indicated by healing gallery), high humidity (70%–100%) and an ambient temperature ranging from 37°C to 41.5°C.

The average duration of the total therapy is 17.5 days (SD 3.5).

The current study did not distinguish between particular treatment-combinations due to limited information on the exact treatment components per patient at the time of data analysis. An intervention might, for example, consist of one individual consultation for back pain prevention, one participation in nutrition advice group, 14 group exercise therapy sessions, eight massage therapy sessions, six mudpacks, six balneotherapeutic radon applications, three group consultations for back pain prevention, eight underwater massages, 11 hydrotherapeutic exercise sessions. Additional or alternative sessions or therapeutic approaches are at the discretion of the responsible spa physicians.

Patients diagnosed with a RMD prior to their visit to the Gastein valley for spa therapy treatment are eligible for inclusion in the radon registry and are recruited by participating spa centre physicians. Following informed consent, standardized paper questionnaires are completed by participants directly before (baseline), directly after as well as 3, 6 and 9 months after multimodal spa therapy including low-dose radon treatment. Questionnaires collect data on sociodemographic characteristics, pain, functional ability and quality of life.

Medical employees from the respective facilities hand over the first two questionnaires in person and send out the questionnaires for the last three timepoints. Questionnaires are then returned to the spa centres using a return envelope. Personal information that would allow for conclusion on the participant’s identity is removed from the data by the medical employees of the spa facilities. The pseudonymized data are then handed over to the employees of the Gastein Research Institute. There, data are manually entered into a digital database by a research assistant.

At the time of analysis for the current manuscript, the radon indication registry comprised 561 subjects with completed questionnaires at all time points of whom 187 had AS; 49 had RA, 61 had KOA, 16 had HOA, 292 had BP, 11 had PsA and 8 had FBM. Participant’s initial recruitment took part between September 2015 and May 2021.

In the current study, current pain in general in rest and motion was evaluated by means of 11-point (NRS) with the anchors being 0 = no pain and 10 = worst pain imaginable. Numeric rating scales (NRS) have demonstrated excellent reliability for the measurement of musculoskeletal pain.

The main independent variable of interest was the timepoint of survey completion. *A priori* determined covariates included age (in years), sex (men/women) and body mass index (BMI; BMI = weight [kg]/height [m]^2^) due to their already established influence on pain [25–27]. To be able to generate RMD specific estimates, the categorical variable “type of disease” was included as covariate distinguishing between AS; RA, KOA, HOA, BP, PsA and FBM.

Descriptive statistics adequate for the metric properties of concerned variables were used to characterize the sample in terms of age, sex, BMI and type of RMD at baseline (i.e., directly before the intervention) and to describe pain in rest and motion for each timepoint and RMD.

To explore the association of timepoint of measurement with a) pain in rest and b) pain in motion while adjusting for age, sex, BMI and type of RMD, two linear regression models were computed. Two-way interactions between timepoint of measurement and type of RMD were explored in both models. After each model, the Stata command “margins” was used to produce age, sex and BMI standardized estimates and their 95% confidence interval (CI) for pain in rest and motion at each timepoint and for each RMD.

*p*-values ≤ 0.05 were considered statistically significant. A change of 1 on the NRS was considered clinically relevant [28].

## Results

The sample had a mean age of 55 years, and 275 (49%) were women. More than 65% of the subjects had a BMI larger than the upper normal value (i.e., 25.0) resulting in an average BMI of 26.8. The unadjusted pain severity in rest and motion at baseline and at the subsequent timepoints of measurement were different across the different types of RMD. The largest difference between baseline pain (in rest and motion) and pain 9 months after therapy was seen in patients with FBM. The smallest difference was found for patients with AS. Table 1 shows 1) the characteristics of the study sample and 2) the unadjusted pain scores directly before, directly after as well as 3; 6 and 9 months after spa therapy including low-dose radon for the complete sample and for each type of RMD.

**TABLE 1:** Characteristics of study sample (*N* = 561) and unadjusted pain scores directly before, directly after and 3; 6 and 9 months after multimodal spa therapy (Salzburg, Austria 2023).

Age, mean (SD) 54.54 (8.85)					
Women, *n* (%) 275 (49.02)					
Body mass index, mean (SD) 26.77 (4.27)					
	Directly before intervention	Directly after intervention	3 months after intervention	6 months after intervention	9 months after intervention
Type of musculoskeletal disease (RMD)	Pain in rest^a^, mean (SD)				
Ankylosing spondylitits (*n* = 187)	3.95 (2.16)	2.23 (1.74)	2.46 (1.83)	2.86 (1.97)	3.36 (2.06)
Rheumatoid arthritis (*n* = 49)	3.99 (2.08)	2.58 (1.98)	2.14 (1.47)	2.62 (1.63)	2.70 (1.85)
Knee osteoarthritis (*n* = 61)	2.39 (1.85)	1.37 (1.18)	1.71 (1.40)	1.66 (1.40)	1.75 (1.30)
Hip osteoarthritis (*n* = 16)	3.66 (2.06)	1.56 (1.62)	1.69 (1.45)	2.06 (2.11)	1.88 (1.70)
Back pain (*n* = 229)	3.54 (1.91)	1.79 (1.51)	2.08 (1.63)	2.33 (1.79)	2.53 (1.93)
Psoriatic arthritis (*n* = 11)	4.55 (0.82)	2.68 (0.90)	3.41 (2.22)	3.96 (2.20)	3.64 (1.55)
Fibromyalgia (*n* = 8)	6.00 (1.87)	3.44 (1.97)	3.13 (2.89)	3.44 (2.43)	3.50 (2.20)
All RMD (*n* = 561)	3.56 (2.06)	1.99 (1.64)	2.20 (1.72)	2.50 (1.87)	2.76 (1.97)
	**Pain in motion^a^, mean (SD)**				
Ankylosing spondylitits	3.90 (2.06)	2.44 (1.71)	2.35 (1.71)	2.87 (1.92)	3.27 (2.08)
Rheumatoid arthritis	5.10 (1.88)	3.17 (2.04)	3.14 (1.92)	3.45 (1.69)	3.55 (2.13)
Knee osteoarthritis	4.35 (2.17)	2.52 (1.88)	3.03 (2.02)	2.84 (1.83)	2.96 (1.88)
Hip osteoarthritis	4.31 (1.96)	2.94 (1.68)	2.03 (1.31)	2.91 (1.63)	2.78 (1.92)
Back pain	4.13 (1.89)	2.07 (1.45)	2.56 (1.86)	2.65 (1.85)	2.87 (2.06)
Psoriatic arthritis	5.31 (1.75)	3.59 (1.20)	3.73 (2.07)	3.73 (1.60)	4.14 (1.76)
Fibromyalgia	7.13 (2.31)	4.56 (1.82)	3.31 (2.52)	3.38 (2.55)	3.69 (2.03)
All RMD	4.23 (2.04)	2.43 (1.71)	2.61 (1.86)	2.85 (1.87)	3.10 (2.06)

^a^ Measured on 11-point numeric rating scale evaluating current pain (0 = no pain; 10 = worst pain imaginable).

Independent of the type of RMD, significant improvements in scores for pain in rest and pain in motion were seen at all follow-up timepoints when compared to baseline. Improvements were largest directly after the intervention (i.e., B −1.65 [95% CI −1.86; −1.44] for pain in rest and B 1.80 [95% CI −2.02; −1.58] for pain in motion). Improvements sustained until 9 months and were clinically relevant until 6 months after intervention for pain in rest and until 9 months after intervention for pain in motion (Table 2). The interaction between timpoint of measurement and type of RMD was not signifcantly associated with pain in rest or motion.

**TABLE 2:** Changes in pain severity in rest and motion after multimodal spa therapy in patients with musculoskeletal diseases^a^ (Salzburg, Austria 2023).

	Changes in pain severity (References = directly before intervention) B [95% CI]	
	In rest	In motion
Timepoint of measurement		
Directly after intervention	−1.65 [−1.86 −1.44]* ^b^	−1.80 [−2.02; −1.58]* ^b^
3 months after intervention	−1.43 [−1.65; −1.22]* ^b^	−1.61 [−1.83; −1.39]* ^b^
6 months after intervention	−1.14 [−1.35; −0.92]* ^b^	−1.37 [−1.59; −1.15]* ^b^
9 months after intervention	−0.88 [−1.09; −0.67]*	−1.13 [−1.35; −0.91]* ^b^

^a^ Results of multivariable linear regression adjusted for age, sex, BMI, and type of RMD (i.e., ankylosing spondylitis, rheumatoid arthritis, knee osteoarthritis, hip osteoarthritis, back pain, psoriatic arthritis, fibromyalgia).

^b^ Clinically relevant change (i.e., >1).

*Statistically significant at *p* ≤ 0.05.

**FIGURE 1:**
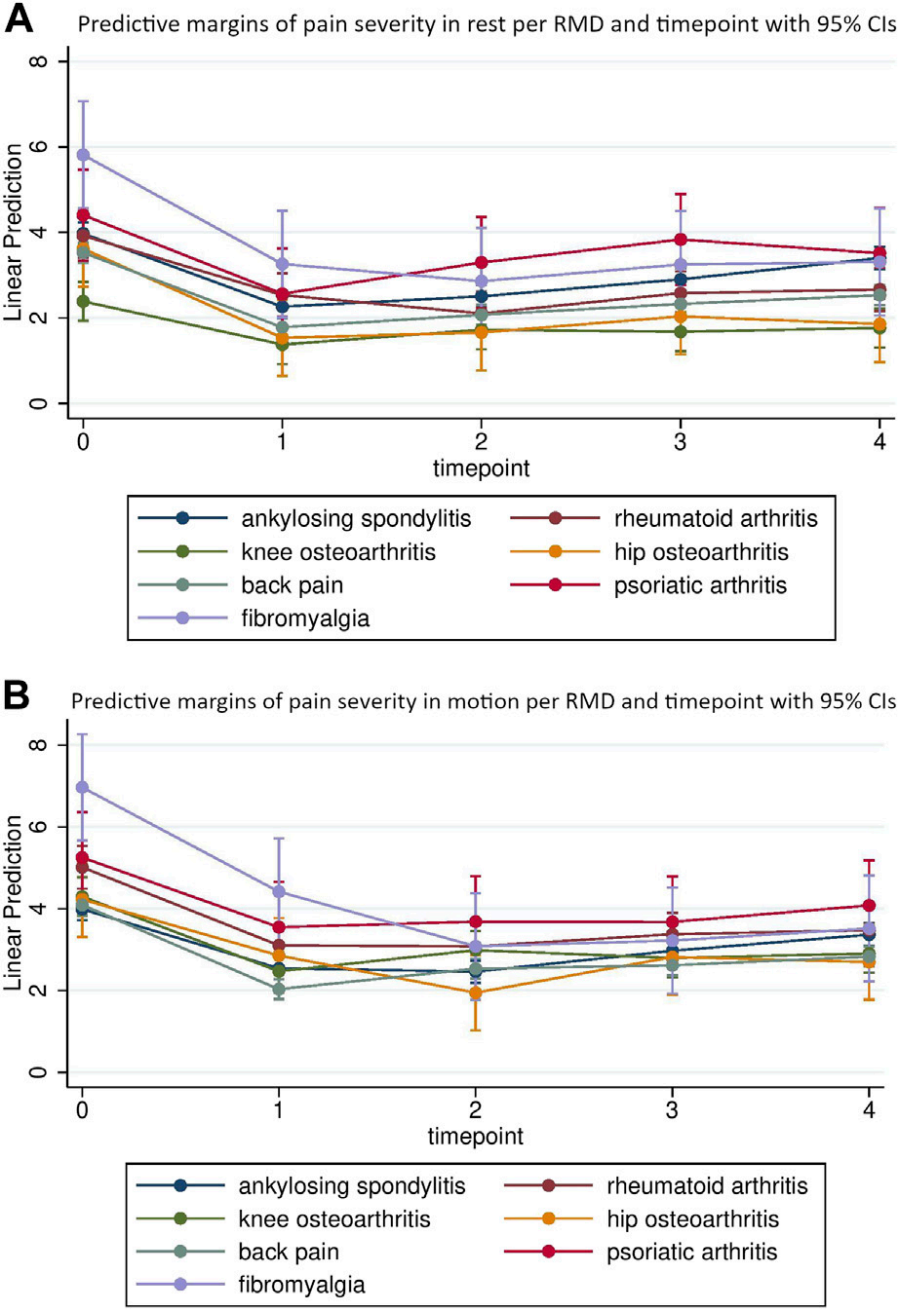
Age, sex and BMI standardized estimates for pain in rest **(A)** and motion **(B)** directly before (t0), directly after (t1) and 3 (t2), 6 (t3) and 9 (t4) months after multimodal spa therapy for each musculoskeletal disease (Salzburg, Austria 2023).

The course of pain alterations was different for each individual disease. For example, the pain in rest for patients with FBM was 5.81 [95% CI 4.56; 7.07] at baseline, 3.26 [95% CI 2.01; 4.51] directly after intervention and 3.31 [95% CI 2.06; 4.56] after 9 months. In patients with AS, predicted pain scores in rest returned towards baseline scores more rapidly (i.e. 3.97 [95% CI 3.71; 4.24] at baseline, 2.26 [95% CI 2.00; 2.52] directly after intervention and 3.40 [95% CI 3.14; 3.66] after 9 months. Similar patterns were observed in the prediction of scores for pain in motion. Table 3 provides the predicted pain scores in rest and motion for each disease and timepoint. Figures 1A, B illustrate the course of pain severity in rest and motion for each disease and timepoint.

**TABLE 3:** Age, sex and BMI standardized pain scores in rest and motion for selected musculoskeletal diseases after multimodal spa therapy (Salzburg, Austria 2023).

	Timepoint of measurement				
	Directly before intervention	Directly after intervention	3 months after intervention	6 months after intervention	9 months after intervention
Type of musculoskeletal disease (RMD)	Pain in rest^a^ margin (95% CI)				
Ankylosing spondylitits	3.97 (3.71; 4.24)	2.26 (2.00; 2.52)	2.50 (2.24; 2.76)	2.90 (2.64; 3.16)	3.40 (3.14; 3.66)
Rheumatoid arthritis	3.92 (3.42; 4.43)	2.53 (2.03; 3.04)	2.10 (1.60; 2.61)	2.58 (2.07; 3.09)	2.66 (2.16; 3.17)
Knee osteoarthritis	2.39 (1.93; 2.84)	1.38 (0.92; 1.83)	1.72 (1.27; 2.18)	1.68 (1.22; 2.13)	1.77 (1.31; 2.22)
Hip osteoarthritis	3.62 (2.74; 4.50)	1.53 (0.65; 2.42)	1.66 (0.78; 2.54)	2.03 (1.15; 2.92)	1.85 (0.97; 2.74)
Back pain	3.53 (3.29; 3.76)	1.78 (1.55; 2.01)	2.07 (1.84; 2.31)	2.33 (2.09; 2.56)	
Psoriatic arthritis	4.41 (3.34; 5.47)	2.56 (1.50; 3.63)	3.30 (2.23; 4.36)	3.83 (2.77; 4.90)	3.51 (2.45; 4.60)
Fibromyalgia	5.81 (4.56; 7.07)	3.26 (2.01; 4.51)	2.86 (1.61; 4.11)	3.25 (2.00; 4.50)	3.31 (2.06; 4.56)
	**Pain in motion^a^ margin (95% CI)**				
Ankylosing spondylitits	3.99 (3.71; 4.26)	2.54 (2.27; 2.81)	2.46 (2.19; 2.73)	2.97 (2.70; 3.24)	3.36 (3.09; 3.64)
Rheumatoid arthritis	5.02 (4.49; 5.54)	3.10 (2.58; 3.63)	3.08 (2.55; 3.60)	3.38 (2.85; 3.90)	3.48 (2.95; 4.00)
Knee osteoarthritis	4.30 (3.83; 4.77)	2.47 (2.00; 2.94)	2.98 (2.51; 3.50)	2.79 (2.32; 3.26)	2.90 (2.43; 3.37)
Hip osteoarthritis	4.23 (3.31; 5.14)	2.85 (1.93; 3.77)	1.94 (1.03; 2.86)	2.82 (1.90; 3.73)	2.69 (1.78; 3.61)
Back pain	4.09 (3.85; 4.33)	2.03 (1.79; 2.27)	2.53 (2.29; 2.77)	2.61 (2.37; 2.86)	2.83 (2.58; 3.07)
Psoriatic arthritis	5.25 (4.14; 6.36)	3.55 (2.44; 4.65)	3.68 (2.58; 4.79)	3.68 (2.57; 4.78)	4.08 (2.97; 5.19)
Fibromyalgia	6.97 (5.67; 8.27)	4.42 (3.12; 5.71)	3.08 (1.78; 4.38)	3.22 (1.92; 4.52)	3.52 (2.22; 4.81)

^a^ Measured on 11-point numeric rating scale evaluating current pain (0 = no pain; 10 = worst pain imaginable).

## Discussion

Supporting the hypotheses formulated prior to this study, significant improvements in pain in rest and motion independent of the type of RMD were found after multimodal spa therapy including a radon-therapeutic element until 9 months after the therapy. These improvements were clinically relevant [28] until 6 months after treatment for pain in rest and until 9 months after treatment for pain in motion.

Previous research has shown similar results. In a systematic review and meta-analysis, Falkenbach et al. [17] identified five randomized controlled trials that compared radon therapy (as balneo- or speleotherapy) with a control intervention and concluded that radon therapy has a positive effect on pain in patients with RA, degenerative spinal disease and ankylosing spondylitis. A more recent clinical trial also found superiority in pain reduction when comparing radon baths to tap water baths in patients with chronic back pain, OA, RA and/or AS [29].

To the best of our knowledge, no other study has compared the course of pain after spa-therapy including low-dose radon across different types of RMD in a real-life setting, yet. In the current study, the course of improvement in pain scores was independent of age, sex and BMI and type of RMD. All types of RMD showed an immediate improvement after the intervention but the course of improvement was different for each type. In a lexical analysis and scoping review, Tognolo et al. [30] showed long-term clinical improvement in a variety of health outcomes (including pain) and RMD but pointed out that the wide variety of interventions, methodologies and employed outcome measures hampers comparability between studies and thus also across different RMD.

From a clinical perspective, it seems plausible that treatment responses vary across disease type. On this line, insight into the underlying disease-specific mechanisms of spa-therapy may aid in understanding reason for these differences but additional evidence is needed to gain understanding of the sustained effect on pain and other health parameters found in the current and previous studies [31, 32].

Evidence regarding differences in treatment responses is crucial in advising medical decision makers (i.e., referring physicians or spa physicians concerned with the development of treatment regimens) as well as insurance companies concerned with financial compensation and standardization of treatment regimens of patients with RMD. The current study adds to this evidence. Interestingly, patients with FBM showed the largest improvements in pain severity compared to baseline at 9 months’ follow-up while patients with AS showed the smallest improvement at this timepoint. This pleads for shorter treatment intervals for patients with AS than for patients with FBM. Simultaneously, it highlights the effectiveness of the multimodal spa-therapy treatment approach under study in FBM, a disease that is frequently considered challenging to treat due to its multifactorial nature [33].

Strengths of the study comprise the relatively large study sample with complete data over a period of 9 months, the independence of data collection and solid statistical procedures for the generation of predictions. Importantly, the findings of the current study were able to support existing evidence from controlled environments in a real-life setting and give rise to further research exploring disease specific therapy responses of multimodal interventions.

Several limitations arise from the use of data registries as was done in the current study. The collection of data was neither monitored nor performed by the researchers and potentially relevant confounders (like acute illness or trauma, physical activity and medication use) for the explored association were not available. Moreover, data truncation at follow-up might have occurred resulting in over-or underestimation of the improvement in pain. For example, if patients who enrolled in the study did not complete follow-up questionnaires but had on average a larger improvement in pain than those considered in the current analyses, underestimation of improvement would be the result. On the same line, regression to the mean must be considered as potential reason for the observed change in pain scores. It must also be noted that data on the frequency of treatment before intervention were not systematically collected which might result in biased baseline values and ultimately underestimation of the improvement in first-time participants as those who received the intervention repeatedly likely have better baseline values. This might particularly be the case for patients with AS who are more frequently returning visitors to spa-facilities in the Gastein valley for secondary prevention of pain symptoms. In addition, pre-post observations based on patient reported outcomes are prone for response-shift bias, and results based on such observations should therefore always be considered carefully as improvement in health might be a reflection of individual perception of health rather than actual health [34, 35].

Some of the subgroups of disease type (i.e., FBM and PsA) only included a limited number of patients, which led to wider confidence intervals and thus less accuracy in the estimates for these disease types. However, discussion with local physicians revealed that the number of patients with these disease types is notably smaller than in other groups of RMD revealing that the study is a reflection of the actual clinical situation in participating spas. As the register is still ongoing, future analyses with larger groups of these underrepresented disease types are possible and are being strived for by the authors.

In conclusion, spa therapy including low-dose radon is associated with clinically relevant and significant improvement in patients with various types of RMD. The course of pain severity is different across the various types of RMD and should therefore be considered in the determination of treatment intervals and frequency. The findings of the current study may assist medical decision makers and insurance companies in the determination of such treatment intervals as timing of visits to spa facilities according to disease specific pain courses may result in sustained pain reduction and hence in enhanced quality of life and reduced use of health services.

## Abbreviations

AS, ankylosing spondylitis; BMI, Body Mass Index; BP, back pain; CI, confidence interval; DMARD, disease-modifying anti-rheumatic drugs; EQ, EuroQol; HOA, hip osteoarthritis; HRM, health resort medicine; FBM, fibromyalgia; NRS, numeric rating scale; NSAID, nonsteroidal anti-inflammatory drug; QoL, quality of life; KOA, knee osteoarthritis; RA, rheumatoid arthritis; RMD, rheumatic musculoskeletal disease; RnIR, radon indication registry for the assessment of pain reduction, increase of quality of life and improvement in body functionality throughout low-dose radon hyperthermia therapy; PsA, psoriatic arthritis; VAS, visual analogue scale; YLDs, years lived with disability.
